# Endovascular Interventions Permit Isolation of Endothelial Colony-Forming Cells from Peripheral Blood

**DOI:** 10.3390/ijms19113453

**Published:** 2018-11-02

**Authors:** Vera Matveeva, Mariam Khanova, Egor Sardin, Larisa Antonova, Olga Barbarash

**Affiliations:** Federal State Budgetary Institution “Research Institute for Complex Issues of Cardiovascular Diseases”, 650002 Kemerovo, Russia; khanovam@gmail.com (M.K.); sardines@mail.ru (E.S.); antonova.la@mail.ru (L.A.); olb61@mail.ru (O.B.)

**Keywords:** endothelial colony-forming cells, peripheral blood mononuclear cells, proliferative activity, percutaneous coronary intervention, coronary artery bypass surgery

## Abstract

Background: Isolation of endothelial colony-forming cells (ECFCs) is difficult due to the extremely low concentration of their precursors in the peripheral blood (PB). We hypothesized that mechanical injury to the arterial wall during percutaneous coronary intervention (PCI) or coronary artery bypass grafting (CABG) may increase the release of circulating ECFC precursors and induce their growth in vitro. Methods: PB samples from patients with coronary artery disease were collected before, immediately after, and 24 h after the surgery in the CABG group. In the PCI group, PB was isolated before, immediately after the insertion of the catheter, immediately after balloon angioplasty, and 24 h after the PCI. A mononuclear fraction of PB was isolated and differentiated into ECFCs with the following immunophenotyping and evaluation of angiogenic properties. Results. The obtained cultures corresponded to the phenotype and tube forming potential consistent with ECFCs. The isolation of ECFCs in the PCI group was successful in 75% of cases (six out of eight patients) after catheter insertion and in 87.5% (seven out of eight patients) after the balloon inflation and stent deployment. These cultures had high/medium proliferative activity in contrast to those obtained before or 24 h after the intervention. Conclusions: Mechanical injury during PCI increases the release of ECFC precursors to the PB and, hence, the efficacy of ECFC isolation.

## 1. Introduction

Endothelial progenitor cells (EPCs) represent a minor mononuclear cell population circulating in the peripheral blood (PB) [1]. EPCs are able to support angiogenesis in ischemic tissues [2] and facilitate vascular repair [3]. Current protocols for EPC isolation from PB describe at least two types of EPCs: (1) early EPCs (eEPCs), representing hematopoietic cells with molecular phenotype resembling monocytes; and (2) endothelial colony-forming cells (ECFCs), which belong to the endothelial lineage according to their morphology, gene expression profile, and phenotype [4]. ECFCs are alternatively termed late EPCs or outgrowth endothelial cells.

eEPCs indirectly contribute to neovascularization via the production of pro-angiogenic cytokines, while ECFCs play a major role in angio- and vasculogenesis through direct incorporation into the vessel wall [3] and paracrine secretion of pro-angiogenic factors [5]. These properties highlight the importance of optimizing ECFC isolation for their use in cell therapy or tissue engineering; however, circulating ECFC precursors are extremely rare and the possibility to isolate autologous ECFCs with high proliferative potential is currently limited.

A method for mobilizing endothelial progenitor cells (EPCs) (CD34^+^CD133^+^ phenotype) from bone marrow into PB by the administration of granulocyte colony-stimulating factor (G-CSF) has been described by Powell et al. [6] However, this method does not enrich ECFC precursors, and even reduces their quantity and inhibits their angiogenic properties [7]. Increased circulating EPC counts in response to ischemia in various vascular beds (e.g., after ischemic stroke) [8] or after exercise-induced ischemia in patients with coronary artery disease (CAD) [9] have been reported. However, cells (CD34^+^CD309^+^CD45^−^) detected in these studies were not ECFC precursors. Another study employing the enrichment and depletion of PB mononuclear cells (PBMCs) demonstrated that ECFC progenitors in the blood are limited to the CD34^+^CD133^−^CD146^+^ cell population [10].

Increased ECFC numbers were detected in the PB of patients with an early phase of acute myocardial infarction [11], and the frequency of ECFC colonies was associated with the severity of ischemia [12,13]. Güven et al. showed that the proportion of circulating EPCs and isolated colonies of ECFCs in patients undergoing coronary angiography positively correlates with the severity of stenosis [12]. An increase in the percentage of isolated ECFCs with high proliferative potential was registered in a porcine myocardial infarction model after intracoronary angioplasty [13].

Approaches to increase the yield of circulating ECFC precursors may be improved with a better understanding of their origin, which remains uncertain. In addition to the currently existing concepts of EPCs arising from bone marrow-derived circulating angioblasts [14,15], there is some evidence suggesting that blood contains resident EPCs with different clonogenic potential [16,17,18]. Resident EPCs from the vessel wall may detach and migrate with the blood flow to the site of injury for vessel wall repair.

According to the concept of their vascular origin, resident EPCs may be released from the intima into the blood in response to mechanical injury of the vascular wall during coronary angiography. Studies have reported an association between increased numbers of ECFCs and ischemia-induced processes in the heart during intravascular interventions [12,13]. ECFC release can be triggered either directly by ischemia, or by intravascular endothelial damage induced by a catheter/balloon; however, it remains unanswered which of these two processes is the underlying one.

Thus, studies focusing on the assessment of vascular injury in the absence of acute ischemia may provide new insights into mechanisms of ECFC release. Balloon angioplasty may be a suitable experimental model, as catheter insertion inflicts a purely mechanical injury to the vessel wall, whereas subsequent balloon inflation and stent deployment result in mechanical injury of the arterial wall and have a local ischemic effect on heart vasculature and tissues. On the contrary, coronary artery bypass grafting (CABG) leads to both ischemia/reperfusion injury and mechanical injury of the tissues and vessels.

The aim of this study was to assess the effect of vascular injury (percutaneous coronary intervention (PCI) and CABG) on the efficacy of ECFC release from the blood of patients with coronary artery disease (CAD).

## 2. Results

### 2.1. Morphological Patterns of Obtained Cell Cultures

Here, we cultured a mononuclear fraction of PB obtained from patients who were subjected to PCI, CABG, or a control group (healthy blood donors) to isolate ECFCs. The results were considered negative when ECFC colonies were absent in individual samples of PBMCs within a 21-day culture. Alternatively, results were considered positive when ECFC cultures had been successfully isolated from PBMCs.

**Figure 1:**
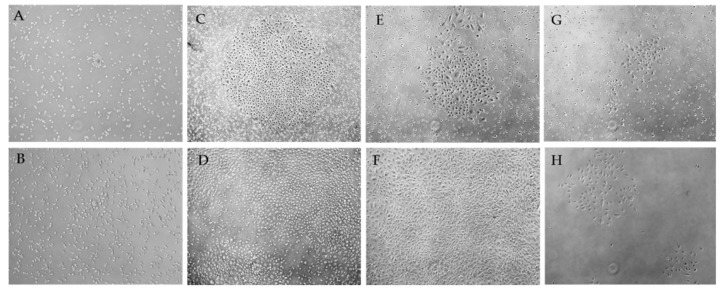
Representative images of mononuclear peripheral blood cultures after 10–15 days (**A**,**C**,**E**,**G**); 16–19-days (**B**,**D**,**F**); 29 days of culture (**H**) (phase-contrast microscopy, 50× magnification). Negative (**A**,**B**) and positive (**C**–**H**) results of ECFC isolation. Images of colonies with high (**C**), medium (**E**), and low proliferative activity (**G**). Expanded ECFCs (**D**) formed the monolayer (**F**). Colonies with over low proliferative activity formed clusters of endothelial cells (**H**).

Negative results were characterized by separated cells of an elongated and rounded shape, weakly or partially adhered to the culture dish (Figure 1). In some cases, single groups of polymorphic cells of an elongated, convoluted, and rounded shape (Figure 1A) were found, which gradually disintegrated and disappeared (Figure 1B).

In the case of positive results, single cells and cell groups with a morphology similar to those described above were detected within 16 days of PBMC culture. After nine to 18 days, the formation of colonies with a cobblestone morphology was documented (Figure 1D,F). Cells were well-adhered to the plastic surface, and had a polygonal or round shape. In different flasks, a number or individual cell colonies with different proliferative activity were formed (Figure 1C,E,G). Proliferating colonies merged with time, gradually displacing the remaining weakly adherent cells, forming a 70–80% confluent monolayer (Figure 1F).

### 2.2. Characterization of Isolated Cells

**Figure 2:**
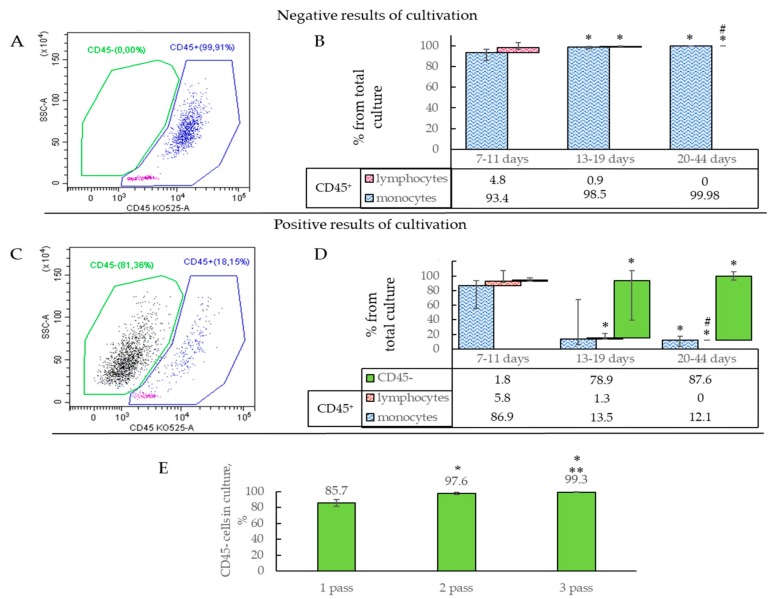
(**A**,**C**) Representative histograms of the distribution of cell populations in resultant cultures based on: (**A**) A negative result (CD45^+^ only) and (**C**) a positive result (two populations CD45^+^ and CD45^−^) (detected by flow cytometry). (**B**,**D**) The composition of cultures at different time points (% of the population of the whole culture, median and 25–75%). (**B**) A negative cultivation result (*n* = 35). (**D**) A positive culture result of ECFC isolation (*n* = 21). * *p* < 0.05 when compared to 7–11 days; # *p* <0.05 when compared to 13–19 days. (**E**) The proportion of the CD45^−^ population in cultures during first to third passages (median and 25–75%); * *p* < 0.05 when compared to one passage; ** *p* <0.05 when compared to two passages.

In order to confirm the endothelial phenotype and assess the purity and composition of the obtained cultures, cells were characterized at different stages of culture using endothelial markers (CD34, CD133, CD31, CD144, KDR, CD146, von Willebrand factor (vWF)) and hematopoietic immune cells (CD3, CD14, HLA DR, CD45). Based on negative or positive results, one or two cell populations were determined in the resulting first passage culture. The significant division was observed with Side Scatter (SSC)/CD45 (Figure 2A,C).

**Figure 3:**
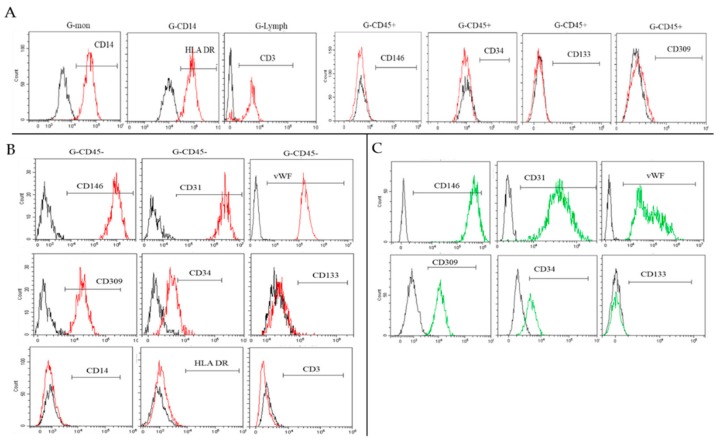
Representative histograms of the antigen expression in different populations: (**A**) CD45^+^, (**B**) CD45^−^, (**C**) HUVECs (flow cytometry).

Each cell population was examined separately with all antigens described above. The proportion of the CD45^+^ population was 99.6–100% in cultures with negative results (Figure 2A,B and Table 1). Positive results were associated with a decreased CD45^+^ population with the expansion of CD45^−^ cells (Table 1 and Figure 2D). In both cases, endothelial and stem antigens were not detected (CD146, CD309, CD133, CD34) in the subpopulation of CD45^+^ cells (Figure 3A).

Importantly, the resultant cell cultures were represented by a mixed culture of monocytes (CD14^+^) and lymphocytes (Figure 2B and Table 1). HLA DR was expressed on monocytes in >50% of cases. Lymphocytes commonly (>85% of cases) consisted of T-lymphocytes (CD3^+^) (Table 1).

Lymphocytes gradually decreased with time in all samples and were undetectable after 20 days of culture. The CD45^+^ population was mainly represented by hematopoietic immune cells, such as monocytes and lymphocytes, whereas after 20 days of culture, it exclusively consisted of monocytes (Figure 2B and Table 1).

During the initial culture, a progressive increase in numbers of CD45^−^ cells (from 1.8% to 87.6%) was observed when the positive results had been confirmed (Figure 2D, green column). Notably, actively proliferating CD45^−^ cells were aggressive in culture. Owing to high adhesion and flatness, these cultures were outgrowing and quickly replacing less adhesive CD45^+^ cells. Before the first passage (at 70–80% confluence), the proportion of CD45^−^ cells was approximately 78.8–91.7%, whilst subsequent passages exhibited a further increase in CD45^−^ cells (on average 97.6% and 99.3% for the second and third passages, respectively) (Figure 2E). In accord, CD45^+^ cells gradually decreased in number and were fully eliminated by the third passage.

The CD45^−^ population had a stable phenotype and was homogeneous in all samples and at all culture time points. CD45^−^ cells had an increased expression of CD146 and CD31, average expression of CD309, and produced vWF in 89.9–95.5% (Table 2). There was no CD133 expression on their membrane. Importantly, a small number of cells (0.1–9.1%) was positive for CD34 (Table 2 and Figure 3B). The CD45^−^ population did not express markers of hematopoietic immune cells CD3, CD14, or HLA DR (Figure 3B). In Table 2 and Figure 3C, human umbilical vein endothelial cells (HUVECs) were characterized using the same markers for comparison.

**Figure 4:**
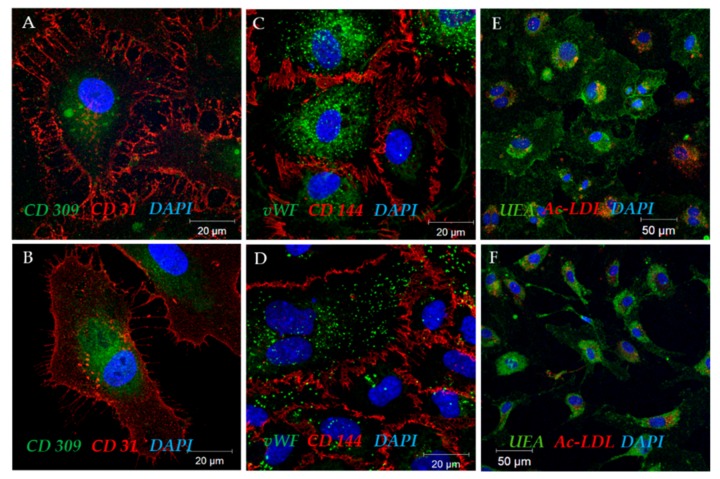
Representative confocal images of CD45^−^ colonies (**A**,**C**,**E**) and HUVEC (**B**,**D**,**F**).

Confocal images (Figure 4) further confirmed the flow cytometry results. CD31 and CD309 receptors (Figure 4A,B) were detected on the surface of both HUVECs and CD45^−^ cells. Intercellular contacts were clearly visualized by the presence of CD144, a cell adhesion protein typical of vascular endothelium (Figure 4C,D). The Weibel-Palade bodies (Figure 4C,D; a bright, clearly delineated green glow) have been determined, as well as diffuse and mesh vWF clusters inside HUVECs and CD45^−^ cells.

Thus, we suggest that obtained CD45^−^ colonies represent ECFCs because they have a similar morphology and phenotype consistent with mature endothelial cells (CD146^+^CD31^+^CD144^+^CD309^+^vWF^+^CD34^+/−^CD133^−^).

### 2.3. Functional Properties of the Selected ECFC Populations

Functional properties of the obtained ECFCs were compared to HUVECs commonly regarded as a standard of mature endothelial cells with proliferative activity.

#### 2.3.1. Uptake of Acetylated Low-Density Lipoproteins and Lectin Binding

We documented the ability of both ECFCs and HUVECs to uptake acetylated low-density lipoproteins (Ac-LDL) and lectin binding (Figure 4E,F), as a feature corresponding to endothelial cells and cultures [19,20,21]. However, it was also reported that all phagocytes may uptake Ac-LDL (specifically neutrophils, monocytes, and various macrophage types) [2]. Thus, the positive test on the uptake of Ac-LDL demonstrates that isolated endothelial cells have a phagocytic ability, but this test will also be positive for all phagocytes.

#### 2.3.2. ECFCs Angiogenic Activity In Vitro

**Figure 5:**
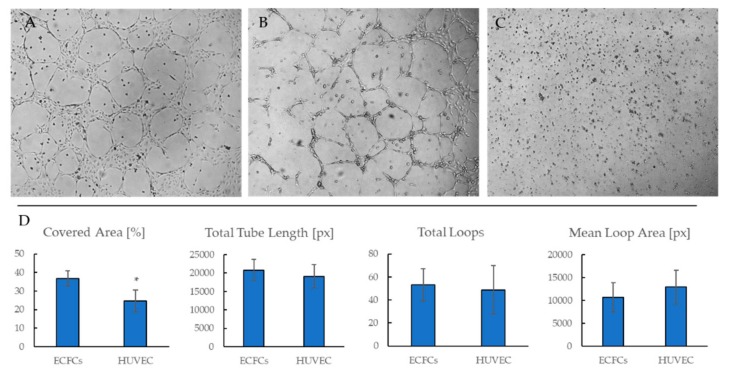
Representative phase-contrast images of ECFCs (**A**), HUVECs (**B**), and CD45^+^ cells (**C**) during a 16-h culture in Matrigel, 50×. **D**. Semi-quantitative image analysis of a tube formation assay (*n* = 5 images per group). * *p* < 0.05.

Similar to HUVECs, isolated ECFC cultures formed capillary-like structures in Matrigel (Figure 5A,B), whereas CD45^+^ cells did not (Figure 5C). Quantitative evaluation of capillary-like structures did not reveal significant differences between HUVECs and ECFCs (Figure 5D), which testified to tubule-forming, angiogenic properties of ECFCs being comparable to mature endothelial cells.

#### 2.3.3. Proliferative Activity of ECFCs

**Figure 6:**
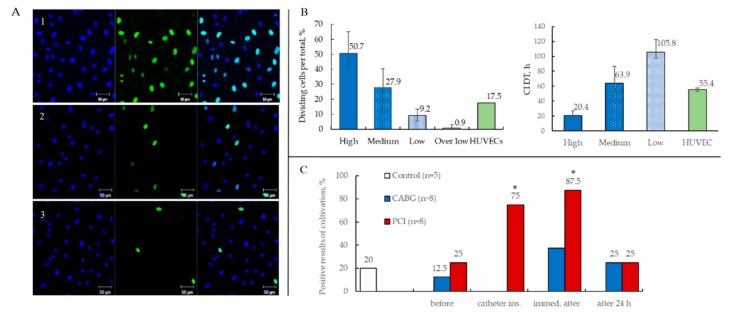
(**A**) Representative confocal images of cultures with different proliferative activity (blue indicates all cell nuclei (DAPI), green indicates dividing nuclei (Alexa Fluor 488), 20×). (**B**) Categorization of ECFC cultures according to their proliferation rate (dividing nuclei per total nuclei (%) and cell index doubling time (CI DT (h)) into four groups. HUVEC proliferation rate is shown for comparison (Mean with range). (**C**) The relative number of positive ECFC cultures results at blood sampling time points in patients undergoing CABG and PCI (* *p* < 0.05 when compared to pre-intervention results).

ECFC cultures demonstrated variable proliferative activity (Figure 6A). In some flasks, cells actively proliferated and reached confluence in three to four days, whereas certain flasks required much more time to reach confluence under equal conditions. We also documented endothelial cell clusters which contained only a few cells and did not proliferate at all (Figure 1H).

We used two approaches for the evaluation of proliferative activity of ECFCs and HUVECs: (1) calculation of the proportion of dividing nuclei during 6-h culture with 5-ethynyl-2′-deoxyuridine (EdU) and (2) count of cell index doubling time (CI DT) utilizing the xCELLigence RTCA DP instrument (Figure 6B).

Based on their proliferation activity, we divided all ECFC cultures into four groups: high (avg. CI DT = 20.4 h); medium (avg. CI DT = 63.9 h); low (avg. CI DT = 105.8 h); and over low, which formed endothelial cell clusters and did not proliferate (Figure 6B). For the fourth passage of HUVECs, the CI DT value was 55.4 h on average, which corresponded to the medium ECFC group. Therefore, our results are consistent with the hypothesis proposed by Ingram et al. suggesting a hierarchy of EPCs circulating in human PB based on their clonogenic and proliferative potential [1].

#### 2.3.4. Results of ECFCs Culture in Different Patient Groups

PB samples were collected from patients who underwent coronary artery bypass grafting (CABG) at three time points: before, immediately after, and 24 h after surgery. Similarly, PB samples were harvested from patients undergoing PCI: (1) before intervention, (2) after the catheter insertion, (3) immediately after balloon inflation and stent deployment, (4) and 24 h after the PCI. Blood samples harvested before PCI represented a baseline for ECFC precursor numbers in CAD patients. Additionally, we collected PB from healthy volunteers (*n* = 5), representing a control group.

ECFCs were rarely obtained from PB samples collected before CABG (one out of eight (12.5%) patients) or PCI (two out of eight (25%) patients) (Figure 6C). The isolation of ECFCs was successful in 75% of cases (six out of eight patients) in the PCI group after catheter insertion and in 87.5% (seven out of eight patients) after the balloon inflation and stent deployment, which significantly exceeded the number of positive results before PCI. Additionally, 24 h after the PCI, the number of successful ECFCs isolation decreased to 25% (two out of eight patients). Notably, there was no statistical difference between positivity for ECFC colonies in cultures between pre-PCI CAD patients and conditionally healthy blood donors. In particular, one out of five healthy volunteers (20%) had ECFC colonies, whereas one or two out of eight patients before intervention yielded positive ECFC cultures (12.5–25%) (Figure 6C).

ECFC cultures were isolated in only three cases (37.5%) from the CABG group immediately after the surgery (Figure 6C). Furthermore, 24 h after the CABG, only 25% of cases (two out of eight patients) had positive results of cultivation. There were no differences in this patient group at different time points.

Thus, the highest number of positive results was obtained during PCI.

**Figure 7:**
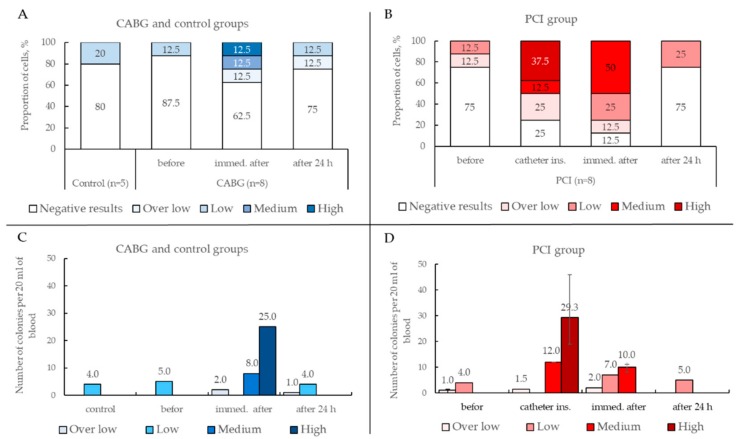
(**A**,**B**) Proliferative activity of ECFC cultures in depending time point harvested blood. (**C**,**D**) Number of ECFC colonies depending on the time point and their proliferative activity.

In this study, we evaluated the level proliferative activity of ECFC cultures in depending time point harvested blood in patients undergoing CABG (Figure 7A) and PCI (Figure 7B). Half of the ECFC cultures (50%) obtained from the blood collected immediately after PCI (balloon inflation and stent deployment) displayed medium or high proliferative activity (Figure 7B), whereas only a quarter of ECFC cultures (25%) exhibited medium or high proliferative activity if the samples were collected immediately after CABG (Figure 7A).

Before and 24 h after PCI or CABG, ECFCs were not commonly isolated or had low and over low proliferative activity. Strikingly, both PCI and CABG groups before and 24 h after surgery showed either no positivity for ECFC or yielded ECFCs with low and over low proliferative activity. Notably, ECFCs isolated from healthy blood donors were also characterized by low proliferation.

Thus, our results demonstrate that PCI leads to the highest yield of actively proliferating ECFC.

To maximize the ECFC yield, in this study, we used Kolbe`s modified protocol with enrichment [22]. According to this method, initial culture lasted seven days, was performed using collagen-coated flasks, and was subsequently followed by culture in fibronectin-coated flasks. Barclay et al. suggested that fibronectin can serve as a good scaffold for ECFC adhesion and expansion, yet can also promote the differentiation of monocyte-derived cells that can mimic EC by phenotype, and may potentially contaminate ECFC cultures [3]. A seven-day long culture on a collagen matrix enables the elimination of monocyte-derived endothelial-like cells. The importance of the seven-day passage is determined by the fact that emerging colonies with high (and possibly medium) proliferation activity give rise to clones, which improves cell expansion, as showed by Kolbe et al. Additionally, the seven-day passage may provide clearance from contaminating hematopoietic cells. Following Kolbe`s protocol in our study resulted in a high yield of colonies with high proliferative activity (19 to 49 colonies from 20 mL blood, depending on the study group) (Figure 7C,D).

These findings are consistent with those presented by Kolbe et al. [22] and significantly exceed those by Ingram et al. [1]. However, during cell passage, there is a risk of ECFC precursor loss, especially those with low and over low proliferative activity. In this study, we isolated four to five colonies with proliferative activity, and none of these showed significant differences regardless of the study group (Figure 7C,D).

### 2.4. Detection of Various EPC Phenotypes and ECFC Precursors in PB and PBMCs

We then tested the hypothesis that the flow cytometry analysis of PB or PBMCs could detect ECFC precursors and therefore predict a positive ECFC yield upon culture. We studied cell populations described in the literature as ECFC or EPC precursors in the PB or PBMCs. The following combinations of markers were studied: CD34^+^ (relative amount (%) among lymphocytes); CD34^+^CD45^−^ (% among CD34^+^); CD34^+^CD45^−^CD133^+^, CD34^+^CD45^−^CD146^+^, and CD34^+^CD45^−^CD309^+^ (% among CD34^+^CD45^−^); and ECFC precursors with the phenotype CD34^+^CD45^−^CD146^+^CD133^−^ (% of CD34^+^CD45^−^). The phenotyping data were grouped according to the culture results (whether or not positive ECFC cultures were obtained) (Table 3).

The analysis did not reveal an association between culture results and relative content of the studied cell populations. We did not detect CD45^−^CD34^+^CD146^+^ and CD45^−^CD34^+^CD133^−^CD146^+^ cells in the PB and PBMCs, which, according to the literature, correspond to the ECFC precursor phenotype. The population of CD34^+^CD45^−^CD309^+^ cells was also not detected.

## 3. Discussion

In spite of controversial opinions, the clinical relevance of EPCs in regenerative medicine is undisputable [23], with multiple interventional studies currently registered at ClinicalTrials.gov. Increasing amounts of effort are being placed on developing vascular grafts with an endothelialized luminal surface, which can interact with the local environment since endothelial cells (ECs) play a significant role in various physiological processes, including hemostasis, thrombosis, smooth muscle cell proliferation, vascular tone etc. [24]. Autologous ECFCs used as a substrate during cell therapy of ischemic areas, the regeneration of injured vascular endothelium, or the pre-seeding of tissue-engineered vascular/valve prosthesis could be of great therapeutic benefit for patients with cardiovascular disease (CVD). However, we and others demonstrate that ECFC precursors are infrequent in the blood of healthy individuals or patients with CVD [3,10,25]. The findings presented here may help to increase the effectiveness of ECFC isolation and culture. PCI is a common diagnostic and treatment procedure in CAD patients. We believe that in patients who are subjected to PCI, blood sampling during the procedure can significantly facilitate the isolation and expansion of autologous ECFCs for subsequent cell therapy or their pre-seeding on the surface of vascular grafts. Our results show that PCI leads to a high yield of actively proliferating ECFC.

For the use of ECFCs in regenerative medicine and tissue engineering, an important point is the possibility of increasing the cell mass, which is directly related to the proliferative activity of cultures. The cultures obtained in our study had variable proliferative potential, whilst during the PCI procedure (insertion of a catheter followed by balloon inflation), the probability of obtaining ECFC cultures with high and medium proliferative potential was significantly higher.

A feature of ECFCs with high and medium proliferative activity is their active expansion followed by the replacement of hematopoietic cells, which enables the former to achieve a purity of more than 99% by the third passage. Taking into account this feature, obtaining autologous ECFC cultures for tissue engineering is possible without additional purification methods (such as cell separation or sorting), by increasing the number of passages and controlling the purity of the cultures by flow cytometry.

Following the concept of the vascular origin of ECFCs, we tested both the possibility of intravascular interventions to increase the likelihood of positive ECFCs isolation and the influence of ischemia on this process. CAD patients undergo PCI for diagnostic and therapeutic purposes. It involves two stages: (1) the insertion of a catheter, which causes mechanical injury of the arterial wall; and (2) the balloon inflation followed by stent deployment, which is additionally associated with short-term local ischemia. Open heart surgery is accompanied by severe mechanical damage to various tissues, including blood vessels, and leads to local (and general) ischemic effects on the heart (and the body as a whole). Thus, the time point after the catheter insertion during PCI is a control for testing mechanical damage in the absence of ischemia, and the next time point after the balloon inflation and stenting serves as a test for combined mechanical and ischemic effects. The subsequent culture of isolated cells demonstrated variable numbers of ECFC precursors in the PB, allowing us to expand some of the ECFC colonies. Immediately at the stage of catheter insertion and stenting, the percentage of positive results of culture was higher than before or 24 h after the procedure. There were no significant differences between time points with the mechanical (catheter insertion) and the combined effect (balloon inflation and stenting). Consequently, intravascular intervention increases the release of ECFC precursors, whilst the ischemic effect on these processes is less substantial.

A similar tendency was observed during CABG, but the observed differences were not statistically significant. This may be explained by the release of ECFC precursors into the area of extensive tissue and vessel damage, as well as their adhesion on the contour surfaces of the artificial circulation pumping system. We do not exclude the possible effect of ischemia on the release of EPC into the blood; however, according to our results, we believe that the main mechanism of EPC release into the blood during PCI is associated with the mechanical impact of the catheter on the vessel wall.

There is evidence suggesting that ECFCs originate from vessels [2,4,16,17,18]. Ingram et al. [16] showed the presence of the complete EPCs hierarchy within the vessel wall (arterial and venous vessels), including cells with high clonogenic potential. Doubting the prevailing concept suggesting the inability of differentiated mature endothelial cells to proliferate, Ingram et al. [16] successfully isolated HUVECs and human aortic endothelial cells from the vascular wall, and these cells possessed high proliferative activity and colony formation ability. Subsequent studies confirmed the presence of resident EPCs in the vascular wall [17], in agreement with the hypothesis of a vasculogenic zone within the wall of adult blood vessels containing EPCs [18]. This concept seems to be logical, since the presence of EPCs in the vascular wall allows a self-sufficient system of vascular integrity and self-healing to be maintained. These data do not deny the value of bone marrow as a possible source of EPCs under certain conditions (such as fetal period or bone marrow stem cell transplantation [14]); instead, they demonstrate that the vascular wall is one of the major sources of EPCs in an adult.

Evidence for the vascular origin of ECFCs also includes the similarity of their morphology, phenotype, genotype, and ultrastructure to mature endothelial cells [4,26]. In our study, the ECFC population also retained the stable phenotype of mature endothelial cells (CD146^+^CD31^+^CD144^+^CD309^+^vWF^+^CD133^−^CD34^+/−^) in all samples and during the whole period of culture and was uniform in composition. In addition, the resultant cultures had endothelial cell properties, including angiogenic activity, and the ability to uptake Ac-LDL, bind lectin, and express vWF.

The ability to predict the isolation of ECFCs from the whole PB or PBMCs would be cost-effective. However, flow cytometry did not allow us to detect cell populations that were putative ECFCs precursors (CD34^+^CD45^−^CD146^+^, CD34^+^CD45^−^CD309^+^, and CD34^+^CD45^−^CD146^+^CD133^−^). In addition, percentages of CD34^+^, CD34^+^CD45^−^, and CD34^+^CD45^−^CD133^+^ populations did not significantly differ depending on the culture results, which is understandable, since the first two populations are too large to detect ECFCs among their predecessors, and the latter population, according to literature, does not contain them [10]. The same approach we used in flow cytometry did not detect ECFC precursors (CD34^+^CD45^−^146^+^, CD34^+^CD45^−^CD309^+^, and CD34^+^CD45^−^CD146^+^CD133^−^). This may be explained by the insufficient number of detected events (for such rare cell population) and the lack of CD34 antigens on the membrane of circulating precursors of ECFCs which mobilized into the blood after the PCI. Endothelium in the veins and arteries of an adult was shown to contain mainly CD34^−^ cells [27] and only a small number of CD34^+^ cells, the so-called tip cells. CD34^+^ endothelial cells demonstrated enrichment for biological function related to angiogenesis and migration, whereas CD34^−^ cells were enriched for functions related to angiogenesis and proliferation [28,29,30].

The proportion of CD34^+^ endothelial tip cells in both the vessel wall [31,32,33] and in vitro is controlled by similar mechanisms [29]. It was also shown that phenotypes of tip cells (CD34^+^) and stalk cells (CD34^−^) in sprouting vessels are reversible and a small subset of CD34^+^ cells remains present in subsequent passages of primary endothelial cell cultures and also in immortalized endothelial cell lines [34]. Our data also demonstrated the presence of a minor CD34^+^ cell population in both ECFC and HUVEC cultures. These findings indirectly confirm the vessel wall origin of ECFCs.

Thus, taking into account the vascular source of ECFCs in the case of intravascular interventions on the arteries, it is likely that the precursors of ECFCs were mostly CD34^−^ populations of endothelial cells with the presence of a minor CD34^+^ population.

## 4. Materials and Methods

### 4.1. Study Population and Design

The study design was approved by the Local Ethical Committee of the Research Institute for Complex Issues of Cardiovascular Diseases (ID 657459, approved 28 October 2016). All patients provided written informed consent before the recruitment.

A total of 16 male patients with CAD referred either to elective coronary artery bypass grafting or PCI (angiography + stenting) were recruited in the study. A brief description of the patient groups is presented in Table 4. The inclusion criteria were as follows: (1) age from 50 to 70 years, prior cardiac surgeries, or PCI. The exclusion criteria were as follows: (1) age over 70 years, (2) acute ischemic processes, (3) concomitant neoplastic and hematological diseases, (4) active inflammation, (5) autoimmune diseases, and (6) postinfarction cardiosclerosis.

The control group included five conditionally healthy male volunteers aged from 34 to 51 years.

**Figure 8:**
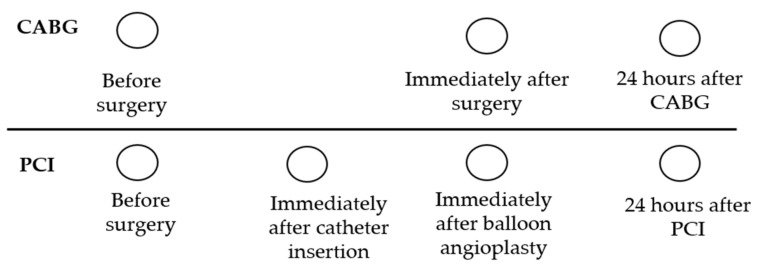
The scheme of time points for harvesting PB in different patient groups.

In this study, 20 mL of peripheral venous blood samples were taken using a 20 mL sterile disposable syringe containing heparin in all patients at control points. PB samples were collected from patients who underwent coronary artery bypass grafting (CABG) at three time points: before, immediately after, and 24 h after surgery. Similarly, PB samples were harvested from patients undergoing PCI: (1) before intervention, (2) after the catheter insertion, (3) immediately after balloon inflation and stent deployment, (4) and 24 h after the PCI (Figure 8).

**Figure 9:**
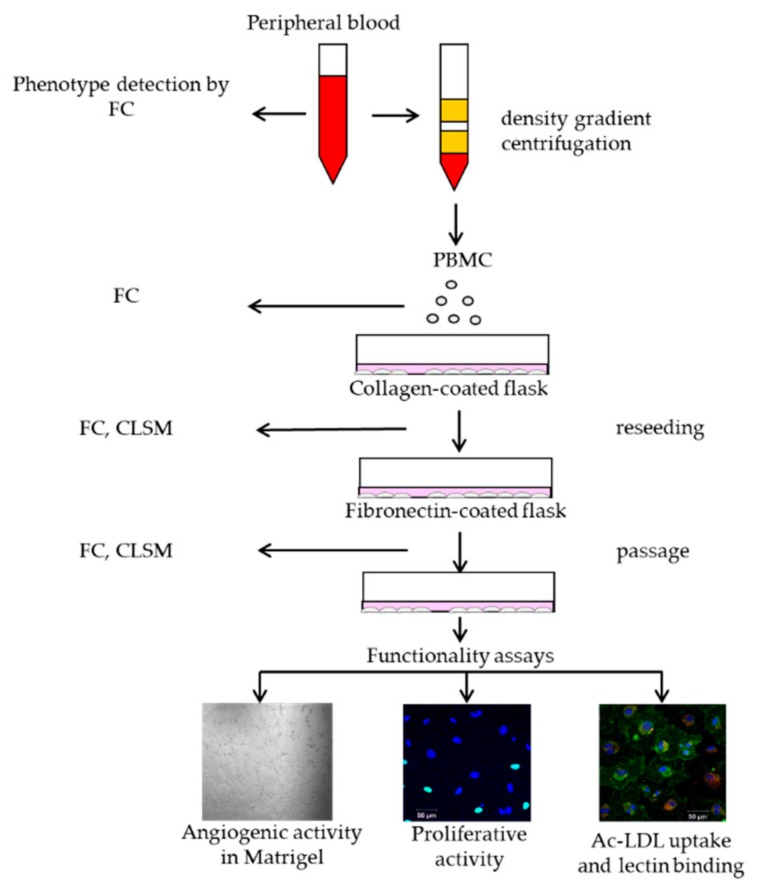
Stages of culture of PBMCs and studies of obtained cultures. (Explanations: FC-Flow Cytometry, CLSM-Confocal Laser Scanning Microscopy).

The PBMCs were isolated using Histopaque density media 1077 (Sigma, St. Louis, MO, USA) according to the manufacturer’s instructions. The cells obtained from the interphase were washed twice with phosphate buffered saline (PBS) followed by centrifugation. We performed isolation and enrichment ECFCs using the modified protocol by Koble et al. [22]. Briefly, cells were resuspended in EGM-2 MV (Lonza, Verviers, Belgium) media supplemented with 5% fetal bovine serum (FBS) (Hyclone, Logan, UT, USA), and plated on collagen-coated 25 cm^2^ flasks. The PBMCs were incubated at 37 °C and in 5% CO_2_. In the first two days, the medium was changed daily to remove nonadherent cells and debris, and then after two to three days. After one week of cultivation, the cells were dissociated with trypsin and reseeded to fibronectin-coated plates for further cultivation in a complete nutrient medium until a 70–80% confluent culture was formed, followed by a passage. Visual control of culture growth was performed daily. We performed the examination of cell phenotype and functional properties of the isolation cultures at various stages of cultivation of PBMCs (Figure 9).

### 4.2. Cell Phenotyping

#### 4.2.1. Flow Cytometry

A total of 100 μL of blood, or 1–2 × 10^5^ PBMCs or 0.5–1 × 10^5^ culture cells, removed from the plates and washed with PBS were taken for staining. Сombined staining was performed with conjugated monoclonal antibodies against human antigens produced by Biolegend (San Diego, CA, USA) (unless otherwise specified): fluorescein isothiocyanate (FITC)—(CD3, CD34, vWF (Abcam, Cambridge, UK), phycoerythrin (PE)—(KDR (BD), CD14), allocicocyanin (APC)—(CD133, CD31), phycoerythrin-cyanine 7 (PC7) CD146, PacificBlue 450 (PB 450) HLA DR, Krome Orange (KrOr) CD45 (BC, Beckman Coulter, Brea, CA).

Whole PB, PBMCs, and cell cultures were allocated according to the manufacturer’s protocols in two panels:

1. CD3, CD14, HLADR, CD45
2. CD34, KDR, CD146, CD133, CD31, CD45

The cell culture with the cobblestone morphology was additionally stained with vWF and CD146.

Blood sample preparation additionally included the lysing of red blood cells with VersaLyse (BC, Beckman Coulter, Brea, CA, USA) and washing with PBS. Fixation and permeabilization of the cells were performed using the IntraPrep (BC) kit when staining intracellular proteins with vWF. The stained samples were resuspended in PBS and analyzed on a CytoFlex flow cytometer (USA) with CytExpert software (version 2.1, Beckman Coulter, CA, USA).

#### 4.2.2. Laser Scanning Microscopy

For subsequent staining with antibodies, the cells were cultured on fibronectin-coated coverslips. Cell staining was performed according to the following protocol: combined staining for von Willebrand factor (vWF) and CD31; vWF and CD144; CD31 and CD309. The cells were fixed for 10 min in a 4% paraformaldehyde solution. To stain intracellular markers (vWF), permeabilization of the cells was additionally performed by treatment with 0.1% Triton X-100 solution for 15 min. To block non-specific binding with the stack, the cells were treated with 1% BSA solution for 1 h. Then, primary antibodies (Abcam, Cambridge, UK) were applied to the samples according to the above-mentioned protocol: anti-CD31 antibody (ab119339), anti-CD144 antibody (ab33168), and anti-CD309 (ab2349), and samples were incubated overnight at +4 °C. Then, the coverslips were incubated at room temperature with secondary antibodies (Abcam, Cambridge, UK): donkey to rabbit IgG Alexa Fluor 555 (ab150074), goat to mouse IgG Alexa Fluor 568 (ab175473), donkey to rabbit IgG Alexa Fluor 488 (ab150073), and antibodies with vWF conjugated to FITC (ab8822). Cell nuclei were stained with fluorescent dye 4′,6-diamidino-2-phenylindole (DAPI) (Sigma (D9542), St. Louis, MO, USA) at a concentration of 10 μg/mL. Then, the samples were mounted with coverslips using ProLong (Life Technologies (P36930), Carlsbad, CA, USA) medium. Control samples were mock-stained with 1% BSA in place of the primary antibodies as for the previously described protocol.

All samples were assessed with the confocal scanning microscope LSM 700 (Zeiss, München Germany).

#### 4.2.3. Proliferative Activity Assessment

Proliferative activity was investigated using two methods: (1) detection of dividing nuclei using fluorescent staining and (2) quantification of cell proliferation in real time using the xCELLigence analyzer.

Fluorescent staining of dividing nuclei was performed using the Click-iT Plus EdU Alexa Fluor 488 Imaging Kit (C10637, Invitrogen, Carlsbad, CA, USA) according to the manufacturer’s protocol. The EdU exposure time was 6 h. The samples were stained with DAPI nuclear dye (Sigma (D9542), St. Louis, MO, USA) at a concentration of 10 μg/mL and mounted onto slides using ProLong (Life Technologies (P36930), Carlsbad, CA, USA) medium. The samples were analyzed with the confocal laser scanning microscope LSM 700 (Zeiss, Germany). Positive stains (green) were counted in 10 randomly chosen fields of vision, and the number of positively stained (green color) cells and their ratio to the total number of cells in the field of view were measured.

We evaluated the proliferative activity of ECFCs utilizing the xCELLigence RTCA DP instrument for noninvasive electrical impedance monitoring. Proliferation capability was defined as cell index doubling time (CI DT), which was calculated automatically in the instrument program. Cells were seeded on 16-well E-plates (2 × 10^4^ cells per well) in duplicate. Impedance was measured over 72 h.

#### 4.2.4. Ability of Cells to Absorb Acetylated Low-Density Lipoproteins (Ac-LDL) and Lectin Binding

A total of 2.4 μg/mL labeled DiI Ac-LDL (Molecular Probes, Grand Island, NY, USA) was injected into the wells with adherent cells and incubated for 2 h at 37 °C. After the removal of the reagent, the cells were fixed with 2% paraformaldehyde for 15 min and incubated for 1 h with lectin Ulex Europaeus Agglutinin-1 conjugated with FITC (FITC-UEA lectin; Sigma) at a concentration of 10 μg/mL. Cell nuclei were stained with DAPI 1.5 μg/mL. Then, the samples were mounted and assessed with the confocal microscope LSM 700.

#### 4.2.5. Angiogenic Activity of Cells In Vitro

Matrigel was added (200 μL) to each well of a 24-well plate for 30 min at room temperature to achieve polymerization. EGM 2 (Lonza) medium with 5% FBS was used in order to set similar conditions (concentration of growth factors) for the cultivation of HUVEC and CFECs. Cells were seeded at 1 × 10^5^ cells per well resuspended in complete medium. The formation of capillary-like structures was observed after 16 h with phase contrast microscopy on the inverted Carl Zeiss microscope.

Semi-quantitative image assessment of the area of capillary-like structures was performed using WimTube (Wimasis Image Analysis, WIMASIS GmbH, Munich, Germany).

#### 4.2.6. Statistical Analysis

Statistical analysis was performed using statistical software package “Statistica 6.0” (Stat-Ease Inc., Tulsa, OK, USA). The data distribution among the samples was estimated using the Kolmogorov-Smirnov test. The normally distributed data were presented as the mean and standard deviation. Nonparametric data were presented as a median and interquartile range (median and 25–75%). Differences between two independent groups were assessed either with the *t*-test or the Mann-Whitney U test, depending on the distribution. Statistical significance was defined as *p* < 0.05 in all tests.

## 5. Conclusions

Mechanical damage caused during PCI releases ECFC precursors with high and medium proliferative potential and enhances the chances to isolate ECFCs from the culture. We suggest that blood sampling during the PCI would be useful in isolating autologous ECFCs for their subsequent expansion and adoptive transfer.

## Figures and Tables

**Table 1:**
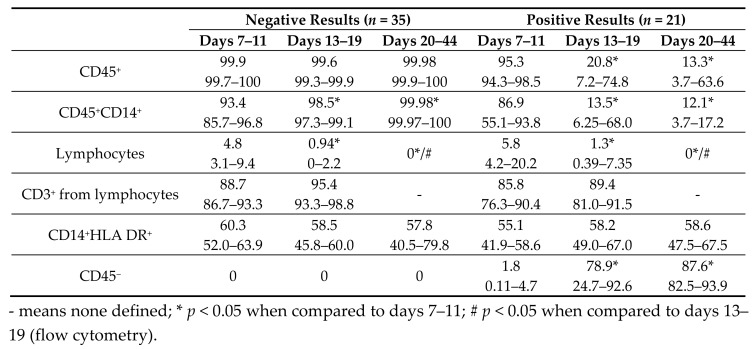
Composition of the cultures at different culture time points.

**Table 2:**
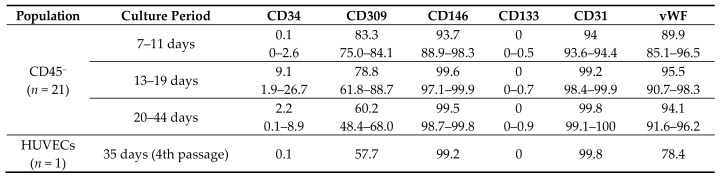
HUVEC phenotype and CD45^−^ population at different culture time points.

**Table 3:** The relative number of cells of interest in PBMCs and whole PB with different culture results.

Results	CD34^+^		CD34^+^CD45^−^		CD34^+^CD45^−^CD133^+^		CD34^+^CD45^−^CD146^+^ CD34^+^CD45^−^CD146^+^CD133^−^ CD34^+^CD45^−^CD309^+^	
	PBMCs	Whole PB	PBMCs	Whole PB	PBMCs	Whole PB	PBMCs	Whole PB
Negative (*n* = 35)	0.05 0.03–0.08	0.04 0.03–0.05	85.9 73.3–93.8	74.2 65.3–86.5	59.1 55.7–62.5	50.0 44.4–70.0	0 0–0	0 0–0
positive (*n* = 21)	0.05 0.04–0.08	0.045 0.04–0.06	86.4 76.0–94.6	66.9 63.4–69.6	60.75 59.3–62.3	54.4 49.3–61.2	0 0–0	0 0–0

**Table 4:** Characteristics of the study population.

	CABG	PCI	*p*-Value
Number of patients	8	8	
Age, years	55.8 (50–64)	61.0 (51–70)	
CAD	CAD	CAD	
PolyVD	25%	25%	
Angina	75%	75%	
Cardiosclerosis	12.5%	62.5	0.039
EF > 50%	100%	100%	
HD III	87.5%	100%	
CHF I	87.5%	75%	
CHF II	12.5%	25%	
Type 2 diabetes	0	25%	
Dyslipidemia	25%	0	
Cerebral atherosclerosis	50%	37.5%	
CPB	62.5%	-	
CPB time, min =	81.4 (67–90)	-	
Aortic cross-clamp time, min	55.4 (43–70)	-	
Duration of procedure	-	38 min (20–80)	
balloon	-	100%	
1 stent/2/3	-	75/12.5/12.5	
complications	12.5% (ACVA)	0	

- means none defined.

## Abbreviations

ECFCs	Endothelial colony-forming cells
PB	Peripheral blood
PCI	Percutaneous coronary intervention
CABG	Coronary artery bypass grafting
CAD	Coronary artery disease
EPC	Endothelial progenitor cell
PBMC	PB mononuclear cell
SSC	Side Scatter
FSC	Forward Scatter
CD	Cluster of differentiation
vWF	von Willebrand factor
HUVEC	Human umbilical vein endothelial cell
HAEC	Human Aortic Endothelial Cells
Ac-LDL	Acetylated low density lipoproteins
EF	Ejection fraction
HD	Hypertonic disease
CHF	Сhronic heart failure
CPB	Coronary pump bypass
PBS	Phosphate buffered saline
FC	Flow Cytometry
CLSM	Confocal Laser Scanning Microscopy
DAPI	4′,6-diamidino-2-phenylindole
